# The prevalence of the third and fourth heart sounds in clinically healthy Holstein cattle

**DOI:** 10.1186/1751-0147-49-12

**Published:** 2007-05-01

**Authors:** Ali Rezakhani, Mehdi Zarifi

**Affiliations:** 1 Department of Clinical Studies, School of Veterinary Medicine, Shiraz University, Shiraz, 71345, Iran

Compared to information available on the third and fourth heart sounds in the horse [1] there is a paucity of information on these sounds in cattle in the veterinary literature, although auscultation is the main step in clinical examination of both the cardiovascular system and the animal as a whole. Most literatures describe bovine cardiac sounds and mention that all four heart sounds may be heard in cattle [2,3]. A review of veterinary literature failed to find information with regard to the prevalence of the third and fourth heart sounds in clinically healthy cattle. This preliminary study was undertaken to find out the incidence of these sounds in clinically healthy cattle.

Three hundred clinically healthy Holstein cattle from 12 dairy farms in Shiraz area were chosen randomly for population to be studied. About 10 minutes after restraining the animals in a stock, the cardiac area was auscultated by two clinicians independently and systematically by a Littman type stethoscope without consulting with each other and then both clinicians listened the heart sounds simultaneously using a sensor stethoscope (Mediron stethoscopes AS, Norway) focusing on the transient heart sounds and cardiac murmurs according to criteria have been presented [4,5]. Before auscultation both sides of cardiac area was palpated for the detection of apex beat. Then stethoscope was placed on the area of the thorax in which apex beat was most clear and attention was focused on the first and second heart sounds and then the stethoscope was moved to the mitral, aortic, pulmonary and tricuspid valves area. The right side of the cardiac area was also auscultated. The age, hear rate and clinical signs of heart disease such as jugular distention and pulsation, edema and thrill were taken into consideration. Animals with the signs of heart diseases were not included in this study.

The results of this study showed that the third heart sound was audible in 8.7% of animals and 29.3% demonstrated the fourth heart sound (Table 1). The prevalence of the fourth heart sound was significantly higher in cattle older than 48 months. None of the calves younger than twelve months had the third heart sound. Both sounds were more prevalent in older ages. There was a reverse correlation between the heart rate and the 4^th ^heart sound. The higher the heart rate the less chance was to hear the 4^th ^heart sounds. The percentage of audible fourth heart sound in the heart rate of higher and lower than 100 BPM was 10.3% and 36% respectively, which was statistically significant (F = 18,5, df = 1, P = 0.000).

**Table 1:** The mean heart rate and prevalence of third and fourth heart sounds

Age groups (month)	The mean of HR (mean ± SE)	S3 (%)	S4 (%)
1–12 (n = 55)	115.36 ± 2.18 ^a^	0 ^a^	3.6 ^a^
13–24 (n = 42)	99.88 ± 2.63 ^b^	2.4 ^a^	4.8 ^a^
25–48 (n = 98)	84.39 ± 2.05 ^c^	18.4 ^b^	37.8 ^b^
>48 (n = 105)	81.52 ± 1.66 ^c^	6.7 ^a^	44.8 ^b^

Total (n = 300)	91.23 ± 2.18	8.7	29.3

Values in the same column followed by a different letter indicate a significant difference (P < 0.05) HR = Heart rate, S3 = third heart sound, S4 = fourth heart sound

'It has been mentioned that the 3^rd ^heart sound can be heard when heart rate increases [6,2]. We could not confirm this finding in our study as the 3^rd ^and 4^th ^heart sounds disappeared as soon as the heart rate accelerated. The majority of the third heart sound had maximal point of intensity over the ventral part of the cardiac area where the first heart sound is clearly audible. The fourth heart sound was audible on the base of the heart

It seems that audibility of the third and fourth heart sounds is partly related to the heart rate. In animals with higher heart rate such as dogs, goats, sheep and pigs, these sounds are not heard and only the first and second heart sounds are audible in these animals. On the other hand these sounds, specially the fourth heart sound, are auscultated easily in over 60% of fit horses [7] because the heart rate is lower in this species of animal and the P-R interval is longer than cattle so the fourth heart sound is separated from the first heart sound. In cattle the heart rate (75.73 ± 9.13) [8] is higher than the horse and lower than dogs, goats, sheep and pigs so it is possible to hear these two sounds in some clinically normal cattle [6]. However, care must be taken to differentiate these sounds and the splitting of the first and second heart sounds. One of the criteria used in this study to differentiate the third heart sound from splitting of the second heart sound was the effect of high heart rate on the third heart sound. Splitting of the second heart sound usually remains unchanged when heart rate increases.

In conclusion this preliminary study showed that the 3^rd ^and 4^th ^heart sounds can be audible in some clinically healthy cattle so they should be considered normal when there is no clinical sign of heart disease.
